# Patient Engagement in Medical Research Among Older Adults: Analysis of the Health Information National Trends Survey

**DOI:** 10.2196/15035

**Published:** 2019-10-29

**Authors:** Lynette Hammond Gerido, Xiang Tang, Brittany Ernst, Aisha Langford, Zhe He

**Affiliations:** 1 School of Information Florida State University Tallahassee, FL United States; 2 Department of Statistics Florida State University Tallahassee, FL United States; 3 College of Human Sciences Florida State University Tallahassee, FL United States; 4 Department of Population Health School of Medicine New York University New York, NY United States

**Keywords:** aging, health care disparities, patient participation, medical informatics

## Abstract

**Background:**

By 2035, it is expected that older adults (aged 65 years and older) will outnumber children and will represent 78 million people in the US population. As the aging population continues to grow, it is critical to reduce disparities in their representation in medical research.

**Objective:**

This study aimed to describe sociodemographic characteristics and health and information behaviors as factors that influence US adults’ interest in engaging in medical research, beyond participation as study subjects.

**Methods:**

Nationally representative cross-sectional data from the 2014 Health Information National Trends Survey (N=3677) were analyzed. Descriptive statistics and weighted multivariable logistic regression analyses were performed to assess predictors of one’s interest in patient engagement in medical research. The independent variables included age, general health, income, race and ethnicity, education level, insurance status, marital status, and health information behaviors.

**Results:**

We examined the association between the independent variables and patient interest in engaging in medical research (PTEngage_Interested). Patient interest in engaging in medical research has a statistically significant association with age (adjusted *P*<.01). Younger adults (aged 18-34 years), lower middle-aged adults (aged 35-49 years), and higher middle-aged adults (aged 50-64 years) indicated interest at relatively the same frequency (29.08%, 29.56%, and 25.12%, respectively), but older adults (aged ≥65 years) expressed less interest (17.10%) than the other age groups. After the multivariate model was run, older adults (odds ratio 0.738, 95% CI 0.500-1.088) were found to be significantly less likely to be interested in engaging in medical research than adults aged 50 to 64 years. Regardless of age, the strongest correlation was found between interest in engaging in medical research and actively looking for health information (*P*<.001). Respondents who did not seek health information were significantly less likely than those who did seek health information to be interested in engaging in medical research.

**Conclusions:**

Patients’ interest in engaging in medical research vary by age and information-seeking behaviors. As the aging population continues to grow, it is critical to reduce disparities in their representation in medical research. Interest in participatory research methods may reflect an opportunity for consumer health informatics technologies to improve the representation of older adults in future medical research.

## Introduction

In the United States, the older population is growing as life expectancy increases, and the *baby boomers*, those born in post-World War II America, reach age 65 years and beyond [1]. By 2035, it is expected that older people will outnumber children and will represent 78 million people in the US population [2]. Generally, increasing life expectancy is considered a positive human development; however, growing older is inherently associated with biological and cognitive degeneration [3]. Deteriorating physical health among older adults is most likely because of an increasing prevalence of chronic conditions such as hypertension, rheumatoid arthritis, heart failure, diabetes, lung disease, cancer, and mood and anxiety disorders [1,4,5].

In the United States, 80% of people aged 65 years and older suffer from multiple chronic conditions [6]. Compared with their younger counterparts, older people have increased rates of comorbidities and complications [5,7]. Although older patients are increasing in number, research has shown that they have been underrepresented in medical research such as clinical trials [8-10]. As such, a clear health disparity exists because older populations are unable to benefit from innovative technologies and treatments that may improve health outcomes, improve quality of life, or reduce their overall disease burden [11].

Interest in enhancing patient engagement in medical research is growing, and patients are increasingly playing the role of active partners invested in better health outcomes [12]. Researchers have been working to tailor research agendas to reduce disparities by developing systems and processes to directly involve patients and communities in research [13-15]. Several studies have identified patient- and community-level barriers to participation, noting a lack of understanding of the benefits of clinical trial participation [16,17]. Therefore, patient engagement in research proactively employs collaborative approaches to inquiry or investigation [18]. When patients are engaged in medical research, the goal is for the patients to clearly understand their role in the research process [19] and to be continuously updated about advances resulting from medical research [20]. Patient engagement has been recognized in the literature for its potential benefits such as improvement in the credibility of results related to higher rates of participation, direct applicability of results, improved translation of results into practice, and advances through the ethical focus on *democratization* of research [21-23]. In addition to participating in clinical trials, patients may engage in medical research by performing very specific roles such as serving on a community advisory board or spokesmanship such as being the *public face* of the project [21]. Informatics researchers have applied patient engagement practices to explore community technology practices, develop technology interventions, and use technology to understand community problems [24].

As more researchers implement participatory research design strategies, informatics professionals may further examine the level of engagement of underserved populations in medical research in comparison with the diffusion of technologies such as direct patient engagement networks as tools for information sharing and participation. In 2016, the US Congress passed the 21st Century Cures Act [25], which authorized US $1.8 billion in funding for the Cancer Moonshot Initiative to accelerate advances in cancer research [26]. The Cancer Moonshot Initiative relies on the recommendations from a Blue Ribbon Panel (BRP) of scientific experts to advise the National Cancer Board on actions the broader community views most able to accelerate research [27]. Reducing cancer disparities across a range of research areas using a cross-cutting theme of patient engagement has been at the forefront of the BRP recommendations [28]. The BRP identified direct patient engagement in cancer research including access to a network of information and tools for data sharing as a promising approach to advancing research [29]. In 2010, the Patient-Centered Outcomes Research Institute (PCORI) was established to fund comparative clinical effectiveness research (CER) with an emphasis on answering questions important to patients by recruiting a large and diverse patient population, thereby assisting patients in making informed health decisions [30]. The PCORI developed a National Patient-Centered Clinical Research Network (PCORnet) to improve the nation’s capacity to conduct CER. This national resource is referred to as a *network of networks* and includes 18 patient-powered research networks [31].

When patients have easily available, accurate, and timely information and use it to make informed choices, they are empowered and experience improved health outcomes [32,33]. In participatory research designs, patients may be both producers and consumers of information with opportunities to build local, grassroots action networks for information dissemination [34]. Despite the intuitive appeal of patient engagement strategies, their efficacy for reducing disparities remains inconclusive [30]. To date, there is little scientific evidence available addressing how interest in engaging in medical research among older adults compares with that of younger generations. Although it is certain that there will be a profound impact on the use of health information and technology by the greater number of older people in the population, an important question for researchers is whether this impact might be larger or smaller because health behaviors and characteristics of older adults are different from those of their younger counterparts. Thus, this study aimed to (1) describe current levels of interest in engaging in medical research in the United States and (2) identify patient-level sociodemographic, behavioral, and health information–seeking characteristics associated with interest in engagement in medical research. Few studies have evaluated patient engagement in medical research using a nationally representative sample, which may help assess the impact of a broadly applicable framework [35]. Devising a baseline of patient engagement in medical research and understanding the sociodemographic characteristics associated with engagement of older people can improve future efforts to increase participation of older adults in medical research and thereby reduce disparities in health outcomes.

## Methods

### Data Collection

We derived data for this analysis from the National Cancer Institute’s Health Information National Trends Survey (HINTS). HINTS is a nationally representative cross-sectional survey administered biennially to adults aged 18 years and older in the United States to monitor the evolution of health information and communication. Each version of HINTS includes slightly different survey questions. We used HINTS 4 Cycle 4 data collected between August and November 2014 via self-administered mailed questionnaires. The survey response rate was 34.4%. Additional HINTS 4 methodology details have been described elsewhere [36].

The deidentified HINTS 4 Cycle 4 dataset is publicly available [37]. At the time of the analysis, HINTS 4 Cycle 4 was the most recent iteration for which responses were available for the following questions:

More and more, people are getting involved in research in new ways beyond being a research subject. They are partnering with medical researchers to help decide what research is done and how it is done. For example, people can suggest important topics to study or how to report results to the public. This is sometimes called “patient engagement” in research.

1. Have you ever heard about “patient engagement” in medical research?

2. Have you ever engaged in medical research in this way?

3. Would you ever be interested in engaging in research in this way?

This population sample included 3677 respondents. The exclusion criteria for this study were based on responses to age. Respondents were excluded if they had a missing response or a response error (n=182).

### Measures

#### Dependent Variables

The study had 3 key outcome variables related to patient engagement in medical research, which were assessed as awareness (“Have you ever heard about ‘patient engagement’ in medical research?” [PTEngage_Heardof]), past experience (“Have you ever engaged in medical research in this way?” [PTEngage_EverEngaged]), and current interest (“Would you ever be interested in engaging in research in this way?” [PTEngage_Interested]). Answer choices for these variables are *yes*, *no*, or *not sure*.

#### Independent Variables

The following variables were tested for independence and correlation. They are grouped as sociodemographics, health-related variables, and health information behavior variables.

#### Sociodemographics

We examined selected self-reported sociodemographic characteristics including gender (male / female), age (18-34 / 35-49 / 50-64 / ≥65 years), income (<US $19,999 / US $20,000-US $34,999 / US $35,000-US $49,999 / US $50,000-US $74,999 / ≥US $75,000), race and ethnicity (non-Hispanic white / non-Hispanic black / Hispanic / Asian / Other), education (less than high school / high school graduate / some college / college graduate and more), occupation (employed / unemployed / homemaker / student / retired / disabled), marital status (married/living as married / divorced / widowed / separated / single), rurality, and active duty military service. Rurality (yes/no) was determined by HINTS variable *RUC2013*, which uses the 2013 USDA rural / urban designation assigned to the respondent’s mailing address. Active duty military service was recoded to a bivariate (yes / no) using the responses for the following question: “Have you ever served on active duty in the US Armed Forces, military Reserves, or National Guard?” A variable describing emotional support and self-efficacy was included as well. Social support (yes / no) acknowledges having someone with whom to talk about problems and to help with decision making.

#### Health-Related Variables

Potentially important clinical characteristics and health behaviors such as cancer history, general health, health insurance, regular provider, and most recent checkup were included. Respondents were asked about their health behaviors such as whether or not they have a regular health care provider and how long it has been since their last routine checkup. These questions allowed responses of *yes*, *no*, or *not sure*. In addition, respondents were asked to rate their confidence in their own ability to take good care of their health using a 5-point Likert scale with 1 being *completely confident* and 5 being *not confident at all*.

#### Health Information Behavior Variables

Respondents were also asked whether they had ever looked for health information from any source, which was a binary *yes* or *no* response. They were asked to qualify their health information seeking by indicating for whom the information was sought (ie, *self*, *someone else*, or both) and what sources (eg, books, family, internet, and library) were used. In addition, the respondents were asked what technologies they used to exchange digital health information with their provider. The options (ie, email, text message, app on mobile device, video conference, or fax) were presented with binary *yes* or *no* responses. We used the derived MedINfo_Cat variable to categorize the responses (ie, email only, text message only, app on smartphone only, video or social media only, fax only, none, or multiple technologies).

We included the responses to questions related to access to electronic medical records and personal health records. The binary (yes/no) question “As far as you know do any of your doctors or health care providers maintain your medical information in a computerized system?” was included to determine provider users of electronic medical records. In addition, we included responses indicating the importance (very important, somewhat important, and not at all important) for the statement, “You should be able to get to your own medical information electronically.” Finally, we included responses indicating confidence (very confident, somewhat confident, and not confident) to the question “How confident are you that you have some say in who is allowed to collect, use, and share your medical information?”

### Statistical Analysis

We conducted a complete case analysis and used SAS software (SAS Institute Inc), version 9.4 to perform all statistical analyses. First, we evaluated the frequencies of sociodemographics and health information behavior variables (see Multimedia Appendix 1).

Then we used the chi-square test to examine associations between sociodemographic and health information behavior variables with each outcome of patient engagement: awareness (PTEngage_HeardOf), past experience (PTEngage_EverEngaged), and current interest (PTEngage_Interested; see Multimedia Appendix 1). We found the awareness (PTEngage_HeardOf) and past experience (PTEngage_EverEngaged) variables to have little statistically significant association with age (adjusted *P*=.22 and *P*=.88, respectively). Therefore, we focused on examining the association between independent variables with a single outcome of a single dependent variable for patient engagement and current interest (PTEngage_Interested), which had an adjusted *P*=.001 and indicates a statistically significant association with age.

We conducted multivariate logistic regression analyses to examine whether the health information behaviors variables that were significantly associated with patient engagement remained significant when controlling for certain sociodemographic factors. Weighted multivariate logistic regression analyses were used to obtain an odds ratio (OR) and 95% CI (see Multimedia Appendix 2). A full model, which included all the variables of interest, was developed, and backward elimination was used to identify covariates that were significantly correlated and influenced the regression estimates. Then we revised the model to include only primary independent variables that were significant in the initial model. A statistical significance criterion of *P<.*05 was used for all analyses. Owing to missing data, sample sizes for these multivariate analyses ranged from 2463 to 3495.

To account for the HINTS sampling design and calculate nationally representative estimates, we applied SAS survey procedures incorporating the jackknife variance estimation technique and HINTS-supplied survey weights. This study was granted an expedited ethical approval by the Florida State University Institutional Review Board.

## Results

### Sociodemographic Characteristics

We defined 4 age groups as follows: younger adults (aged 18-34 years), lower middle-aged adults (aged 35-49 years), higher middle-aged adults (aged 50-64 years), and older adults (aged ≥65 years). Each of the sociodemographic characteristics differed significantly with respect to our age groups (Table 1). The full results of the Chi-square tests are provided in Multimedia Appendix 1.

Most study respondents were aged between 50 and 64 years (higher middle-aged), married, college educated, white, lived in urban areas, and homeowners and had an annual household income over US $50,000. Older adults tended to be white (80.00%) and less diverse than the other age groups. Younger adults (aged 18-34 years) tended to have higher educational attainment (50.27%, college or higher) than older adults (27.13%, college or higher). Lower middle-aged, higher middle-aged, and older adults had larger married representation (66.95%, 67.82%, and 56.24%, respectively) than younger adults who were predominately single and never married (28.79% married and 67.39% single). Older adults had the highest representation of widowers (24.90%). Among younger adults, a vast majority reported having emotional support (92.69%) or friends and family to talk with about health (89.17%). Similarly, among older adults most reported having emotional support (88.50%) and having friends or family to talk with about health (90.72%). The older adults have served in the military for active duty more than any other age group (23.87%).

**Table 1:** Weighted percentage of individual characteristics by age group.

Variable		Younger adults (aged 18-34 years)	Lower middle-aged adults (aged 35-49 years)	Higher middle-aged adults (aged 50-64 years)	Older adults (aged ≥65 years)	All
**Race and ethnicity^a^ (n=3229), n (weighted %)**						
	Non-Hispanic white	225 (58.19)	333 (62.38)	701 (73.73)	651 (80.00)	1940 (34.05)
	Non-Hispanic black	61 (13.26)	144 (12.53)	215 (10.43)	103 (6.67)	523 (25.30)
	Hispanic	89 (16.76)	176 (19.88)	148 (10.73)	118 (10.50)	531 (23.98)
	Non-Hispanic Asian	27 (7.69)	35 (3.70)	37 (3.69)	23 (2.54)	122 (22.15)
	Other	25 (4.09)	29 (1.51)	44 (1.41)	15 (0.39)	113 (15.59)
**Gender^b^ (n=3460), n (weighted %)**						
	Male	141 (50.00)	265 (48.87)	495 (48.03)	468 (43.40)	1369 (48.05)
	Female	323 (50.00)	472 (51.13)	714 (51.97)	582 (56.60)	2091 (51.95)
**Education^a^ (n=3470), n (weighted %)**						
	Less than high school	19 (5.05)	67 (11.87)	97 (12.93)	122 (21.04)	305 (11.62)
	12 years or completed high school	58 (12.50)	105 (19.27)	243 (19.34)	248 (24.32)	654 (18.08)
	Some college	143 (32.17)	192 (26.92)	425 (32.43)	311 (27.51)	1071 (30.02)
	College graduate or higher	245 (50.27)	376 (41.92)	450 (35.30)	369 (27.13)	1440 (40.28)
**Income range^a^ (n=3166), n (weighted %)**						
	<US $19,999	99 (22.53)	120 (13.64)	278 (17.31)	235 (25.33)	735 (19.26)
	US $20,000-US $34,999	67 (12.84)	73 (8.58)	143 (11.62)	189 (21.23)	472 (12.72)
	US $35,000-US $49,999	72 (14.59)	103 (16.15)	150 (12.56)	142 (16.04)	467 (14.74)
	US $50,000-US $74,999	94 (17.90)	123 (16.47)	181 (18.01)	139 (16.05)	537 (17.24)
	≥US $75,000	117 (32.13)	287 (45.16)	362 (40.50)	192 (21.35)	958 (36.03)
**Have health insurance^a^ (n=3446), n (weighted %)**						
	Yes	369 (82.62)	626 (86.78)	1032 (86.57)	1014 (98.20)	3041 (87.41)
**Have a regular health care provider^a^ (n=3439), n (weighted %)**						
	Yes	227 (47.01)	449 (63.4)	886 (74.42)	855 (81.93)	2417 (64.20)
**How many times did you access your own personal health information online through a secure website or app in the last 12 months?^c^ (n=3438), n (weighted %)**						
	None	312 (68.34)	498 (69.64)	871 (74.33)	844 (81.32)	2525 (74.45)
	1-2 times	72 (16.57)	118 (14.83)	144 (10.58)	107 (10.06)	441 (13.47)
	3-5 times	42 (6.70)	72 (8.96)	88 (7.59)	52 (4.86)	254 (7.21)
	6-9 times	16 (3.09)	24 (3.94)	48 (3.33)	23 (1.87)	111 (3.16)
	≥10 times	21 (5.30)	20 (2.64)	45 (4.16)	21 (1.89)	107 (3.71)
**Seek health information^c^ (n=3463), n (weighted %)**						
	Yes	385 (78.64)	634 (84.53)	994 (82.64)	825 (77.60)	2838 (81.04)
**Interested in medical research^b^ (n=3368), n (weighted %)**						
	Yes	148 (29.08)	216 (29.56)	329 (25.17)	191 (17.10)	884 (23.15)

^a^Statistically significant, *P*<.001.

^b^Statistically significant, *P*<.01.

^c^Statistically significant, *P*<.05.

### Health Information Behaviors

Nearly all older adults (98.20%) reported having health insurance. Similarly, 85.41% of older adults indicated having had a checkup in the past year and 81.93% confirmed having a regular health care provider. With respect to having confidence in their own ability to take care of their health, all age groups selected “very confident” more than any other option (younger adults, 44.25%; lower middle-aged, 47.07%; higher middle-aged, 45.58%; and older adults, 48.41%). Health information–seeking activities were present among all groups. Younger and older adults least frequently sought health information (78.64% and 77.60%, respectively), whereas lower middle-aged and higher middle-aged adults tended to report seeking health information slightly more (84.53% and 82.64%, respectively). Among those who sought health information, all age groups were more likely to report using the internet more than any other method, but a smaller proportion of older adults use the internet to seek health information (46.93%). In addition to the internet, older adults seek health information from their doctor (26.16%), which is a greater proportion than any other age group’s second most popular information channel. Although it was not found to be a statistically significant independent variable, a majority of all age groups used no technology to exchange medical information with health care professionals. Yet, every age group placed a high level of importance on an individual ability to get personal medical information electronically.

### Patient Engagement

Regarding interest in engaging in medical research, younger, lower middle-aged, and higher middle-aged adults indicated interest at relatively the same frequency (29.08%, 29.56%, and 25.17%, respectively), but older adults expressed slightly less interest (17.10%) than the other age groups. A multivariate model was run using interest in engagement in medical research as the dependent variable and the remaining variables as independent variables (see Multimedia Appendix 1). Variables that were significant in the initial model were included in the final model (see Multimedia Appendix 2).

Compared with higher middle-aged adults, older adults (OR 0.738, 95% CI 0.500-1.088) were significantly less likely to be interested in engaging in medical research (Table 2). Many sociodemographic characteristics were not significantly associated with interest in engaging in research, but a few characteristics related to health behaviors and access to health data were found to be statistically significant. When controlling for all other variables, not having a regular health care provider reduces the odds of interest in engaging in medical research (OR 0.643, 95% CI 0.419-0.986). In addition, respondents who accessed their personal health information 6 to 9 times through a secure website or app in the last 12 months were 3 times as likely to be interested in engaging in medical research than those who had not accessed their personal health information. Finally, the strongest correlation was found between interest in engaging in medical research and actively looking for health information (*P*<.001). Respondents who did not seek health information were significantly less likely than those who did seek health information to be interested in engaging in medical research.

**Table 2:** Interest in medical research by sociodemographic characteristics, health behaviors, and information-seeking correlates.

Variable		Odds ratio	95% Wald confidence limits	*P* value
**Age group (years)**				
	Younger, 18-34	1.260	0.729-2.179	.37
	Low middle, 35-49	1.315	0.886-1.951	.12
	High middle, 50-64 (reference)	1	—^a^	—
	Older, ≥65	0.738	0.500-1.088	.046^b^
**Have a regular health care provider**				
	Yes (reference)	1	—	—
	No	0.643	0.419-0.986	.04^b^
**How many times did you access your own personal health information online through a secure website or app in the last 12 months?**				
	None (reference)	1	—	—
	1-2 times	1.161	0.689-1.957	.08
	3-5 times	1.388	0.773-2.492	.44
	6-9 times	3.064	1.523-6.167	.02^b^
	≥10 times	2.586	1.175-5.692	.18
**Seek health information**				
	Yes (reference)	1	—	—
	No	0.253	0.134-0.476	<.001^c^

^a^Not applicable for references.

^b^Statistically significant, *P*<.05.

^c^Statistically significant, *P*<.001.

## Discussion

### Principal Findings

This study reports the prevalence of patients’ interest in engaging in medical research using data collected from the 2014 HINTS. Using these nationally representative data, we were able to explicate relationships between specific sociodemographic characteristics, health behaviors, and information-seeking activities. The key finding from our analyses was that the association of age with interest in engaging in medical research remained significant after adjustment for potential confounders. In addition, we found that having a regular health care provider, accessing your personal health information 6 to 9 times per year, and seeking health information increased the odds of being interested in engaging in medical research.

### Older Adults

Levy and Sidel [38] described social injustice conceptually as the denial of economic, sociocultural, political, civil, or human rights to individuals based on the perception of their inferiority by those with more power or influence. Operating under this definition of social injustice, it is the charge of our society to implement policies and actions to counter injustice [39]. We must ensure conditions under which people can be healthy. Therefore, it is the societal duty to strongly recommend greater investments in aging research and translation of study results into safe, affordable, and universally available applied technologies and treatments [3]. Previous research showed that older adults are often directly (with age criterion) excluded from participation in clinical trials [40]. The result of this study shows that older adults (aged ≥65 years) are less interested in engaging in medical research than middle-aged adults. Opportunities for the engagement in medical research by older populations may serve as a means to advocate and prioritize the needs of older adults to reduce disparities. Researchers may use informatics tools to assess priorities of information tracking systems, institution infrastructure, research infrastructure, navigator and personnel programs, and community partnerships and patient advocates relevant to older adults to increase access to education, screening, and research participation opportunities [41].

### Regular Health Care Provider

In this study, we found that having a regular health care provider significantly increases the likelihood of interest in engaging in medical research. A number of factors could be in play. As many patients rely on their health care providers as their first choice for health information, providers may act as gatekeepers for information about medical research. Physicians may also be researchers and enrich the quality of both services and research studies [42]. However, it should be noted that the strongest arguments against gatekeeping center on the patient’s lack of freedom, lack of choice, and the erosion of patient-doctor trust that springs from the doctor’s prerogative to decide on any referral [43]. Another concept could be that patients with a regular provider are actively treating a disease, and because of this, the patient is more familiar and interested in medical research. For example, for cancer patients, diagnosis represents a communications and information flow between providers and patients to support informed decision making [44]. Diagnosis may include consultations and counseling to determine the best treatments and opportunities to participate in research [44]. Despite the prevalence of the internet, social media, and smartphone apps, patients tend to rely most on physicians as a source of information on cancer and medical research [45].

### Health Information Behavior

Acquiring and making sense of health information is vital to patients making important decisions for their own health and the health of their families [46]. Medicine is an information-intensive enterprise [47]. To make informed choices and navigate within a complex health care system, consumers must have easily available, accurate, and timely information, and they must use it [33]. Moreover, 2 key health information covariates, accessing personal health information 6 to 9 times a year and seeking health information, appear to be correlated with interest in engaging in medical research. Both may be related to patient activation, which refers to empowering patients to play an active role in health care [12,48]. When patients are provided with access to their health information, they tend to have higher levels of satisfaction with their providers, increased understanding of their care, more engagement in health improving behaviors, and improved health outcomes [49]. Consumer health information technologies may help improve provider-to-patient communication, health monitoring, and information access to support self-care [50]. Although older adults are traditionally *late adopters* of technology, many use the internet and mobile devices to seek out health information [51]. Community-engaged health informatics, which combines concepts and methods from biomedical informatics, community-based public health approaches, and community informatics, may present opportunities for informatics advancements in patient engagement in medical research [52]. In addition, with advances in informatics methods such as Generalizability Index for Study Trait [53] and tools such as Visual Analysis Tool of Clinical Study Target Population [54], medical researchers are discovering innovative ways to reduce disparities through improvement in population representativeness of their studies.

### Limitations

This study is limited in 2 ways. First, HINTS does not include questions directly qualifying the respondents’ interest in engaging in medical research. Future research should address this gap to better understand specifically what activities related to patient engagement in medical research were of interest, how they learned about opportunities for patients to engage in medical research, and whether they face any barriers to engaging. Second, as HINTS is a cross-sectional survey, it is not possible to infer causal relationships between variables. Despite these limitations, these findings present an opportunity to further explore differences among age groups, to better understand if specific behaviors related to patient engagement in medical research are of interest to older adults, and to identify how older adults learn about opportunities to engage in medical research. Future research may seek to describe additional factors, such as psychosocial influences, spatial or geographic trends, or diffusion of innovative consumer health information technologies. Such inquiry would benefit from a survey tool specifically designed to assess the level of patient interest in engaging in medical research.

### Conclusions

The results of this study demonstrate that disparities exist among older adults with respect to interest in engaging in medical research. Advances in consumer health informatics within existing health systems and research agendas show promise. Future studies should focus on identifying optimal information systems for engaging patients and rigorously examining the impact of these tools for patient engagement in medical research. Specifically, there is a clear need for both methodological and practical research on patient engagement in medical research that translates to improved health outcomes and reduced disparities.

## Appendix

Multimedia Appendix 1 Weighted percentage of individual characteristics by age group.

Multimedia Appendix 2 Interest in medical research by sociodemographic characteristics, health behaviors, and information-seeking correlates.

## Abbreviations

BRP: Blue Ribbon Panel
CER: clinical effectiveness research
HINTS: Health Information National Trends Survey
NIH: National Institutes of Health
OR: odds ratio
PCORI: Patient-Centered Outcomes Research Institute
