# Supplementary material for: Patient Engagement in Medical Research Among Older Adults: Analysis of the Health Information National Trends Survey
Source: J Med Internet Res. 2019 Oct 29;21(10):e15035. doi: 10.2196/15035 (PMC6914241; doi:10.2196/15035)
Supplement: Multimedia Appendix 1 [file jmir_v21i10e15035_app1.pdf]

Multimedia Appendix 1. Weighted percentage of individual characteristics by age group.

Variable		Younger adults (aged 18-34 years)	Lower middle-aged adults (aged 35-49 years)	Higher middle-aged adults (aged 50-64 years)	Older adults (aged ≥65 years)	All
**Race and ethnicity [a] n=3229, n (weighted%)**						
	Non-Hispanic white	225 (58.19)	333 (62.38)	701 (73.73)	651 (80.00)	1940 (34.05)
	Non-Hispanic black	61 (13.26)	144 (12.53)	215 (10.43)	103 (6.67)	523 (25.30)
	Hispanic	89 (16.76)	176 (19.88)	148 (10.73)	118 (10.50)	531 (23.98)
	Non-Hispanic Asian	27 (7.69)	35 (3.70)	37 (3.69)	23 (2.54)	122 (22.15)
	Other	25 (4.09)	29 (1.51)	44 (1.41)	15 (0.39)	113 (15.59)
**Gender [b] n=3460, n (weighted %)**						
	Male	141 (50.00)	265 (48.87)	495 (48.03)	468 (43.40)	1369 (48.05)
	Female	323 (50.00)	472 (51.13)	714 (51.97)	582 (56.60)	2091 (51.95)
**Education [a] n=3470, n (weighted %)**						
	Less than high school	19 (5.05)	67 (11.87)	97 (12.93)	122 (21.04)	305 (11.62)
	12 years or completed high school	58 (12.50)	105 (19.27)	243 (19.34)	248 (24.32)	654 (18.08)
	Some college	143 (32.17)	192 (26.92)	425 (32.43)	311 (27.51)	1071 (30.02)
	College graduate or higher	245 (50.27)	376 (41.92)	450 (35.30)	369 (27.13)	1440 (40.28)
**Marital status [a] n=3452, n (weighted %)**						
	Married	169 (28.79)	418 (66.95)	589 (67.82)	496 (56.24)	1672(53.59)
	Living as married	34 (1.89)	34 (4.35)	39 (3.02)	12 (1.38)	119 (2.75)
	Divorced	17 (1.16)	104 (6.92)	299 (13.18)	169 (11.92)	589 (7.59)
	Widowed	1 (0.01)	15 (1.67)	78 (3.84)	286 (24.90)	380 (5.73)
	Separated	16 (0.76)	28 (1.80)	44 (1.51)	21 (1.55)	109 (1.36)
	Single, never been married	227 (67.39)	139 (18.32)	156 (10.62)	61 (4.01)	583 (28.97)
**Income range [a] n=3166, n (weighted %)**						
	<$19,999	99 (22.53)	120 (13.64)	278 (17.31)	235 (25.33)	735 (19.26)
	$20,000-$34,999	67 (12.84)	73 (8.58)	143 (11.62)	189 (21.23)	472 (12.72)
	$35,000-$49,999	72 (14.59)	103 (16.15)	150 (12.56)	142 (16.04)	467 (14.74)
	$50,000-$74,999	94 (17.90)	123 (16.47)	181 (18.01)	139 (16.05)	537 (17.24)
	≥$75,000	117 (32.13)	287 (45.16)	362 (40.50)	192 (21.35)	958 (36.03)
**Occupation status n=3390, n (weighted %)**						
	Employed	318 (66.67)	549 (77.97)	719 (66.19)	142 (11.93)	1728 (60.18)
	Unemployed	39 (8.59)	61 (6.97)	70 (5.56)	17 (1.22)	187 (6.11)
	Homemaker	36 (3.96)	74 (8.24)	42 (3.99)	47 (6.32)	199 (5.52)
	Student	58 (20.00)	13 (1.59)	3 (0.23)	—	74 (6.67)
	Retired	1 (0.12)	3 (0.45)	171 (13.37)	768 (75.42)	943 (16.46)
	Disabled	5 (0.68)	31 (4.83)	175 (10.65)	48 (5.11)	259 (5.05)
**Active duty [c] n=3404, n (weighted %)**						
	Yes	25 (4.07)	47 (6.64)	120 (9.19)	246 (23.87)	438 (9.39)

**Rent or own your home [a] n=3437, n (weighted %)**					
	Own	160 (30.67)	450 (66.92)	843 (77.93)	818 (78.45)	2271 (60.57)
	Rent	276 (58.07)	268 (31.58)	340 (20.86)	213 (19.65)	1097 (34.94)
	Occupied without paying monetary rent	25 (11.26)	15 (1.49)	15 (1.20)	14 (1.90)	69 (4.49)
**Rurality [a] n=3495, n (weighted %)**						
	Urban	429 (90.07)	683 (89.03)	1047 (81.12)	899 (78.56)	3058 (85.53)
	Suburban	15 (5.44)	25 (4.91)	57 (6.56)	71 (9.47)	168 (6.28)
	Rural	23 (4.50)	35 (6.07)	116 (12.31)	95 (11.98)	269 (8.18)
**Have emotional support [c] n=3400, n (weighted %)**						
	Yes	418 (92.69)	629 (86.36)	1026 (89.28)	891 (88.50)	2964 (89.41)
**Have friends/family to talk about health n=3403, n (weighted %)**						
	Yes	412 (89.17)	642 (88.60)	1048 (89.44)	926 (90.72)	3028 (89.35)
**Own ability to take care of health [a] n=3413, n (weighted %)**						
	Completely confident	130 (29.77)	143 (17.77)	239 (19.87)	172 (17.39)	684 (21.92)
	Very confident	213 (44.25)	340 (47.07)	536 (45.58)	492 (48.41)	1581 (46.06)
	Somewhat confident	101 (23.01)	214 (29.79)	330 (27.91)	308 (29.53)	953 (27.19)
	A little confident	14 (2.86)	26 (3.72)	59 (4.57)	45 (3.53)	144 (3.63)
	Not confident at all	2 (0.11)	11 (1.65)	28 (2.08)	10 (1.13)	51 (1.19)
**Have health insurance [a] n=3446, n (weighted %)**						
	Yes	369 (82.62)	626 (86.78)	1032 (86.57)	1014 (98.20)	3041 (87.41)
**Have a regular health care provider [a] n=3439, n (weighted %)**						
	Yes	227 (47.01)	449 (63.4)	886 (74.42)	855 (81.93)	2417 (64.20)
**How long it has been since their last routine checkup [a] n=3444, n (weighted %)**						
	Within past year	268 (55.80)	490 (66.69)	903 (73.55)	899 (85.41)	2560 (68.27)
	Within past 2 years	74 (14.82)	122 (15.90)	140 (12.50)	70 (7.00)	406 (13.18)
	Within past 5 years	63 (16.50)	52 (7.35)	56 (4.52)	25 (2.21)	196 (8.59)
	5 or more years ago	42 (8.39)	44 (5.93)	69 (6.14)	19 (2.13)	174 (6.09)
	Do not know	14 (2.60)	15 (2.25)	21 (1.99)	21 (2.19)	71 (2.28)
	Never	3 (1.88)	12 (1.88)	15 (1.30)	7 (1.06)	37 (1.59)
**As far as you know do any of your doctors or health care providers maintain your medical information in a computerized system? [b] n=3412, n (weighted %)**						
	Yes	408 (85.37)	653(89.76)	1096 (92.04)	978 (94.73)	3135 (89.83)
**Importance of ability to get to your own medical information electronically [a] n=3377, n (weighted %)**						
	Very important	342 (72.11)	521 (71.09)	854 (72.90)	584 (56.22)	2301(69.35)
	Somewhat important	99 (22.99)	160 (21.80)	260 (21.47)	277 (26.13)	796 (22.82)
	Not at all important	20(4.90)	44 (7.10)	68 (5.63)	148 (17.65)	280 (7.83)
**How many times did you access your own personal health information online through a secure website or app in the last 12 months? [c] n=3438, n (weighted %)**						

	None	312 (68.34)	498 (69.64)	871 (74.33)	844 (81.32)	2525 (74.45)
	1-2 times	72 (16.57)	118 (14.83)	144 (10.58)	107 (10.06)	441 (13.47)
	3-5 times	42 (6.70)	72 (8.96)	88 (7.59)	52 (4.86)	254 (7.21)
	6-9 times	16 (3.09)	24 (3.94)	48 (3.33)	23 (1.87)	111 (3.16)
	≥10 times	21 (5.30)	20 (2.64)	45 (4.16)	21 (1.89)	107 (3.71)
**Used ... to exchange medical information with a health care professional** [a] **n=3411, n (weighted %)**						
	Email only	18.0	11.6	15.3	9.8	477 (14.19)
	Text message only	0.7	2.3	1.9	0.5	41 (1.41)
	App on a smartphone or mobile device	0.6	1.4	2.5	0.3	40 (1.22)
	Social media or video conference only	0.7	0.8	1.3	0.5	38 (0.85)
	Fax only	1.4	2.3	2.4	1.8	71 (1.94)
	None	65.0	69.4	66.4	82.1	2400 (69.46)
	Multiple modes selected	13.7	12.2	10.2	5.0	344 (10.93)
**Seek health information** [c] **n=3463, n (weighted %)**						
	Yes	385 (78.64)	634 (84.53)	994 (82.64)	825 (77.60)	2838 (81.04)
**Looked for information about health or medical topics from...** [a] **n=2463, n (weighted %)**						
	Print	10 (2.53)	30 (6.09)	76 (7.49)	116 (17.84)	232 (7.36)
	Family	9 (5.33)	8 (1.40)	20 (2.65)	27 (5.34)	64 (3.53)
	Friend/coworker	6 (1.51)	14 (3.23)	12 (1.79)	14 (1.77)	46 (2.11)
	Doctor or health care provider	25 (8.73)	63 (12.29)	144 (15.62)	193 (26.16)	425 (14.40)
	Internet	269 (80.71)	441 (75.51)	582 (70.27)	355 (46.93)	1647 (70.93)
	Other	5 (1.20)	9 (1.48)	19 (2.18)	16 (1.96)	49 (1.65)
**Heard of medical research n=3374, n (weighted %)**						
	Yes	44 (9.76)	108 (14.21)	157 (13.15)	149 (14.81)	458 (12.66)
**Interested in medical research** [b] **n=3368, n (weighted %)**						
	Yes	148 (29.08)	216 (29.56)	329 (25.17)	191 (17.10)	884 (23.15)
**Ever engaged in medical research n=3365, n (weighted %)**						
	Yes	8 (1.70)	14 (1.75)	29 (2.27)	31 (2.18)	82 (1.94)

[a]Statistically significant, *P*<.001.
[b]Statistically significant, *P*<.01.
[c]Statistically significant, *P*<.05.
