# Supplementary material for: Patient Engagement in Medical Research Among Older Adults: Analysis of the Health Information National Trends Survey
Source: J Med Internet Res. 2019 Oct 29;21(10):e15035. doi: 10.2196/15035 (PMC6914241; doi:10.2196/15035)
Supplement: Multimedia Appendix 2 [file jmir_v21i10e15035_app2.pdf]

Multimedia Appendix 2. Interest in medical research by sociodemographic characteristics, health behaviors, and information-seeking correlates.

Variable		Odds ratio	95% Wald confidence limits	*P* value
**Age group (years)**				
	Younger, 18-34	1.260	0.729-2.179	.37
	Low middle, 35-49	1.315	0.886-1.951	.12
	High middle, 50-64 (reference)	1	—[a]	—[a]
	Older, ≥65	0.738	0.500-1.088	.046[b]
**Ethnicity**				
	White (reference)	1	—[a]	—[a]
	Black	0.876	0.531-1.446	.51
	Hispanic	1.182	0.766-1.822	.40
	Asian	0.810	0.302-2.173	.64
	Other	1.075	0.409-2.830	.82
**Gender**				
	Male	0.978	0.729-1.314	.89
	Female (reference)	1	—[a]	—[a]
**Education**				
	Less than high school	1.111	0.554-2.228	.69
	12 years or completed high school	0.944	0.515-1.732	.79
	Some college	0.958	0.685-1.340	.76
	College graduate or higher (Reference)	1	—[a]	—[a]
**Marital status**				
	Married (reference)	1	—[a]	—[a]
	Living as married	0.963	0.474-1.956	.66
	Divorced	1.277	0.779-2.092	.47
	Widowed	0.743	0.465-1.187	.08
	Separated	1.881	0.680-5.230	.21
	Single, never been married	1.041	0.600-1.804	.79
**Have served on active duty in the United States**				
	Yes	1.103	0.678-1.793	.69
	No (reference)	1	—[a]	—[a]
**Income range**				
	<$19,999	0.771	0.407-1.463	>.99
	$20,000-$34,999	0.648	0.377-1.113	.32
	$35,000-$49,999	0.690	0.390-1.218	.57
	$50,000-$74,999	0.789	0.470-1.324	.91
	≥$75,000 (reference)	1	—[a]	—[a]
**Occupation status**				
	Employed (reference)	1	—[a]	—[a]
	Unemployed	1.174	0.485-2.843	.76
	Homemaker	0.699	0.339-1.441	.24
	Student	0.942	0.393-2.260	.80
	Retired	1.401	0.951-2.065	.06
Disabled		1.214	0.598-2.462	.62
**Rent or own your home**				
	Own (reference)	1	—[a]	—[a]
	Rent	2.014	1.407-2.885	.11

	Occupied without paying monetary rent	0.850	0.128-5.647	.59
**Rurality**				
	Urban (reference)	1	—[a]	—[a]
	Suburban	1.613	0.649-4.009	.33
	Rural	1.024	0.549-1.911	.57
**Have health insurance**				
	Yes (reference)	1	—[a]	—[a]
	No	1.126	0.591-2.144	.72
**Have a regular health care provider**				
	Yes (reference)	1	—[a]	—[a]
	No	0.643	0.419-0.986	.04[b]
**How long it has been since last routine checkup**				
	Within past year (reference)	1	—[a]	—[a]
	Within past 2 years	0.923	0.558-1.527	.32
	Within past 5 years	0.850	0.339-2.131	.27
	5 or more years ago	1.285	0.588-2.959	.93
	Do not know	1.440	0.290-7.151	.92
	Never	4.005	0.160-100.288	.43
**Confidence in own ability to take good care of their health**				
	Completely confident	1.243	0.846-1.825	.86
	Very confident (reference)	1	—[a]	—[a]
	Somewhat confident	0.796	0.496-1.277	.17
	A little confident	0.935	0.395-2.209	.59
	Not confident at all	2.659	0.299-23.645	.38
**Have emotional support**				
	Yes (reference)	1	—[a]	—[a]
	No	1.257	0.623-2.535	.52
**Have friends/family to talk about health**				
	Yes (reference)	1	—[a]	—[a]
	No	0.852	0.454-1.600	.62
**As far as you know do any of your doctors or health care providers maintain your medical information in a computerized system?**				
	Yes (reference)	1	—[a]	—[a]
	No	1.523	0.651-3.564	.33
**You should be able to get to your own medical information electronically?**				
	Very important (reference)	1	—[a]	—[a]
	Somewhat important	0.521	0.333-0.813	.16
	Not at all important	0.656	0.297-1.450	.82
**How many times did you access your own personal health information online through a secure website or app in the last 12 months?**				
	None (reference)	1	—[a]	—[a]
	1-2 times	1.161	0.689-1.957	.08
	3-5 times	1.388	0.773-2.492	.44
	6-9 times	3.064	1.523-6.167	.02[b]
	≥10 times	2.586	1.175-5.692	.18
**Seek health information**				
	Yes (reference)	1	—[a]	—[a]
	No	0.253	0.134-0.476	<.001[d]

[a]—: Not applicable for references

[b]Statistically significant, *P*<.05.

[c]Statistically significant, *P*<.01.

[d]Statistically significant, *P*<.001.
